# Dresden PTSD treatment study: randomized controlled trial of motor vehicle accident survivors

**DOI:** 10.1186/1471-244X-6-29

**Published:** 2006-07-06

**Authors:** Andreas Maercker, Tanja Zöllner, Hans Menning, Sirko Rabe, Anke Karl

**Affiliations:** 1 University of Zurich, Department of Psychopathology and Clinical Intervention, Switzerland; 2 Klinik Roseneck Center for Behavioral Medicine, Prien am Chiemsee, Germany; 3 University of Technology Dresden, Biopsychology Unit, Germany; 4 University of Southampton, School of Psychology, UK

## Abstract

**Background:**

We translated, modified, and extended a cognitive behavioral treatment (CBT) protocol by Blanchard and Hickling (2003) for the purpose of treating survivors of MVA with full or subsyndromal posttraumatic stress disorder (PTSD) whose native language is German. The treatment manual included some additional elements, e. g. cognitive procedures, imaginal reliving, and facilitating of posttraumatic growth. The current study was conducted in order to test the efficacy of the modified manual by administering randomized controlled trial in which a CBT was compared to a wait-list control condition.

**Methods:**

Forty-two motor vehicle accident survivors with chronic or severe subsyndromal posttraumatic stress disorder (PTSD) completed the treatment trial with two or three detailed assessments (pre, post, and 3-month follow-up).

**Results:**

CAPS-scores showed significantly greater improvement in the CBT condition as compared to the wait list condition (group × time interaction effect size d = 1.61). Intent-to-treat analysis supported the outcome (d = 1.34). Categorical diagnostic data indicated clinical recovery of 67% (post-treatment) and 76% (3 months FU) in the treatment group. Additionally, patients of the CBT condition showed significantly greater reductions in co-morbid major depression than the control condition. At follow-up the improvements were stable in the active treatment condition.

**Conclusion:**

The degree of improvement in our treatment group was comparable to that in previously reported treatment trials of PTSD with cognitive behavioral therapy.

**Trial registration:**

ISRCTN66456536

## Background

Annually, more than 125.000 people are severely injured and approximately 6000 die in motor vehicle accidents (MVAs) in Germany [1]. Epidemiologic research in the United States [2,3] and Germany [[4,5] Maercker, Perkonigg, Schmutzer & Brähler, submitted] has confirmed that MVAs are among the most frequent traumatic events that lead to Posttraumatic Stress Disorder (PTSD) in these countries. Recent prospective studies of injured MVA survivors report PTSD prevalences ranging from about 2% [6] to about 18% [7].

Prospective follow-up studies of injured MVA survivors [summarized in [8]] showed that about 50% of initial cases of PTSD remit within the first 6 months post-MVA. Those who continue to be symptomatic beyond 6 months, and especially beyond 12 months, tend to have a chronic course of the disorder with noticeable psychosocial impact [9]. Thus, there is a need for treatment and research on treatment for this population.

Recent meta-analyses and surveys have shown sufficient effectiveness of psychotherapy for PTSD, and cognitive-behavioral treatment in particular [10-13]. Much of this research has either involved male combat veterans who were 10–25 years post-trauma or female survivors of sexual assault who were on average 5–10 years post-trauma. Less research has been conducted with other homogenous trauma-populations such as MVA survivors or survivors of natural disasters. In a randomized controlled trial, Blanchard and associates demonstrated that a manualized CBT program designed for MVA survivors who suffer from PTSD or subsyndromal PTSD is superior to control conditions (i.e. supportive therapy, wait list [14]). They also demonstrated that the achieved PTSD symptom improvements were stable for the CBT condition over two years [14,15].

Cognitive behavioral treatment (CBT) for PTSD usually includes exposure and cognitive restructuring procedures [16]. Analyses showed that both procedures are highly effective [10,11]. During the last decade, specifically elaborated cognitive procedures have been developed and evaluated [17-20]. Recent PTSD treatment research showed however that further treatment techniques applied solely or embedded into new approaches are also highly efficient. Among them are writing assignments [20,21], facilitation of posttraumatic growth [22,23], or social sharing [21]. One goal of the present study was to combine well-established CBT techniques for PTSD treatment (imaginal or in-vivo prolonged exposure and cognitive restructuring) with additional procedures such as writing assignments, social sharing, and facilitation of posttraumatic growth. Based on Blanchard and Hickling's [8] manual on standard CBT for posttraumatic stress in MVA survivors, we developed an advanced CBT manual [24] to include these treatment elements.

The present study was aimed to apply a translated and extended version of this CBT program in German MVA survivors and to test its efficacy and the stability of the improvements 3 months after the treatment.

## Methods

### Experimental design and patient flow

Recruitment of patients was performed at the University of Technology Dresden, Germany, from April 2002 to August 2004. Treatment and 3-month follow-ups on all participants were completed in February 2005. The protocol was approved by the local ethics board and all participants gave written informed consent. After the patients have been recruited and completed the initial assessment, they were matched into dyads based on the initial Clinician Administered PTSD Scale (CAPS) score, diagnosis (full vs. subsyndromal PTSD) and randomly assigned to one of two conditions: (1) a combination of cognitive behavioral treatment (CBT) procedures or (2) a Wait List Control (WLC) condition. The latter was crossed over after the post-assessment to the CBT condition.

Of 239 potential participants who were screened by telephone, 132 were offered an appointment for assessment and 110 completed the assessment. Eleven were excluded on the basis of co-morbid diagnoses (1 with bipolar disorder, 5 with current alcohol or drug abuse or dependence, and 2 with noticeable cognitive impairment secondary to the MVA – as clinically observed by assessors); 44 others were not included in the treatment study because of too few symptoms or too low CAPS score. Sixty-five patients were eligible for treatment and therefore offered treatment. Seventeen refused treatment due to geographical distance. Forty-eight attended at least one treatment session. Data from all of these were used in the intent-to-treat analysis. There were six dropouts (2 from CBT, 4 from Wait List). Thus, 42 participants completed the post-treatment assessment.

### Participants

Our sample included 22 individuals who met DSM-IV criteria for PTSD and 20 individuals with severely symptomatic subsyndromal PTSD [meets criterion A, E and F for PTSD and two of criteria B, C, or D [see [8] or [25] for a description of the utility of this category] with a CAPS score of 30 or higher. Demographic variables for the participants in each of the treatment conditions, and for the treatment drop-outs combined, are presented in Table 1.

**Table 1:** Demographic information on patient completer groups and dropouts

**Variable**	**Condition**	
	**CBT N = 21**	**Wait List N = 21**
Gender (M/F)	2/19	8/13
Age		
M	39.4	41.3
SD	11.4	10.8
Average years of education		
M	12.8	13.2
SD	2.5	2.8
Months since MVA		
M	63.1	49.1
SD	75.2	52.9
Continuing medical treatment after MVA in days:		
in-patient: M	18.1	24.9
out-patient: M	225.1	265.1

The CBT and WLC conditions did not differ significantly in any of the variables presented in Table 1 even though there were more men in the wait list control than in the CBT condition. The two dropouts from CBT condition did not differ significantly from treatment completers on any tabulated variable.

A reliability check on diagnosis of PTSD was made by having a trained graduate student assessor, blind to diagnosis, reassess half of the sample (n = 20) by listening to audio tapes of the initial interview. Kappa for diagnostic agreement was 0.78, p < 0.001. Correlation of the two total CAPS scores was r (n = 20) = 0.94, p < 0.001.

### Therapists

In order to increase the external validity of the study by working with a broad variety of therapists, six therapists (5 female, 1 male) were trained for the present CBT protocol. All were already licensed psychotherapists or in their last year of postgraduate psychotherapy education. They saw participants in the university practice offices.

The first author and three other licensed and experienced therapists supervised the therapists by regular meetings with video tapes or tele-conferences. All therapy sessions were video taped and scored for treatment adherence to our CBT manual by graduate students. The correspondence between raters and corresponding ideal protocols was kappa = 0.63 (s.d. = 0.13, p < .05).

### Measures

Postgraduate students in Clinical Psychology conducted all assessments after the last author had extensively trained each student. To the extent possible, a participant was assessed by the same assessor at all assessments. By instruction of each study participant the assessors were kept blind to treatment condition.

#### Initial assessment

Participants gave a written informed consent at the initial visit. At the initial assessment circumstances of the participant's MVA were taken without going into detail (no exposure to trauma was intended). The participant was assessed using the Clinician-Administered PTSD Scale (CAPS, German version [26]). The CAPS was scored both for the month immediately following the MVA and for the current time. The current CAPS generated two scores, both a categorical diagnosis of either PTSD, subsyndromal PTSD or non-PTSD, and a total score obtained by summing the ratings of frequency and severity of each of the 17 symptoms. The latter was our primary dependent variable.

Next, all participants were assessed for the possible presence of other Axis I disorders through the use of the SCID-I (German version: [27]).

Several questionnaires were also administered to assess current state and provide data comparable to that reported in other studies. These included Impact of Event Scale-Revised (IES-R; German version: [28]), the Beck Depression Inventory (BDI; German version: [29]), the State-Trait Anxiety Inventory (STAI; German version: [30]), and the Posttraumatic Cognitions Inventory (PTCI: [31]; German version: [32]). Furthermore, self-perceived posttraumatic growth was assessed which will be published separately. Also, a psychophysiological assessment was conducted which will be published elsewhere [e.g., [33]]. Participants were paid €15 for completing the initial assessment including the psychophysiological investigation.

#### Post-treatment assessment

At the conclusion of treatment, participants were reassessed. All of the questionnaires were repeated as was the psychophysiological assessment. The CAPS was re-administered as the chief dependent measure and again scored both categorically for diagnosis and for a total symptom score. Participants were again paid an additional €15 for completing this assessment.

#### Three-month follow-up assessment

All of the procedures used at the post-treatment assessment, including the interviews, questionnaires and psychophysiological assessment were repeated. Participants were again paid €15 for completing this assessment.

### Randomization and wait-list procedure

One of the principal investigators (A.K.) matched participants into dyads, based on age, total CAPS score and diagnosis (either PTSD or subsyndromal), and number of comorbid diagnoses and then randomly assigned dyads to conditions and to therapists according to a list of random numbers previously assigned to each participant.

The wait-list control individuals were told that their treatment would be delayed for 2–3 months and that they would need to be reassessed after that interval before beginning their treatment. After the post-treatment assessment, they were all given the CBT if they were still interested in treatment.

### Treatment

In an effort to enhance the external validity, the treatment allowed the therapist a range of 8–12 weekly sessions with an expected mode of 10. Thus, the therapist could end treatment after 8 visits if in his/her judgment maximum benefit had been obtained; on the other hand treatment could be extended up to 12 visits if the therapist believed the extra sessions were necessary. Mean number of visits for CBT was 11.4 (s.d. = 3.2).

The CBT condition combined several behavioral and cognitive procedures which had been manualized [24]. The initial visit consisted of a detailed description of PTSD and its symptoms and discussion of how the specific patient's symptoms fitted the description. Emphasis was placed on helping the patient to understand PTSD as a reaction to trauma, or "to normalize" the patient's view of his/her symptoms. A description of the treatment components was given. Lastly, the patients were asked to write a very detailed description of the MVA and its immediate aftermath, including their thoughts and sensory perceptions, and bring it to the next session.

Three kinds of exposure were emphasized. First was the reading aloud of his/her description of the MVA by the participant. This was done after the second session. Additionally, patients were asked to read it aloud repeatedly (up to three times) at home and record their 'subjective units of distress' (SUDs). This was tapered to once per day when the patient complained of boredom in the task. This procedure was designed to force the participant to learn to reduce cognitive avoidance.

The second form of exposure was in sensu exposure. It followed the standard procedure for prolonged imaginal exposure described by [16]) and consisted of in-sensu imagination of worst moment(s) of the traumatic event. The therapists encouraged the patient to stay with the memory until habituation leads to reduction of subjectively experienced distress. Sequences of this imagination are repeated until the patient reaches stable levels of lowered or disappeared stress.

The third form of exposure was in vivo exposure to fear arousing cues related to traveling by automobile. An individual hierarchy of tasks was constructed with the patient and travel tasks assigned for each week beginning in Week 3. It could range from sitting behind the wheel of the car, starting it, and backing out of the driveway, in very avoidant cases, to driving near the MVA site or under conditions vaguely related to travel conditions at the time of the MVA. Gentle pressure was exerted to have the patient accomplish more each week. In addition, they were urged to use the newly learned relaxation skills to counter the arousal caused by working on travel hierarchy items. Involvement of spouse/significant other for these exercises was encouraged.

Cognitive procedures concerned four domains. First, basic cognitive restructuring [34] comprised instructions to the patients to monitor their thoughts and feelings associated with the accident-related negative emotional states. Patients were taught to identify cognitive fallacies and learned how to dispute them. In addition, they were taught to identify negative self-talk and how to correct it with positive coping self-talk.

Second, specific accident-related cognitions as described in the Ehlers and Clark model [35] of cognitive treatment of PTSD were identified and questioned, e.g., themes of safety ("I will never feel safe again"), over-generalisation of danger ("Traffic is never safe"), and memory disturbances ("I am loosing my mind because of what happened").

Third, special emphasis was given to identify subjective guilt and anger feelings. In case of predominating feelings of guilt or anger somewhat extended cognitive modules were applied to address and dispute these feelings [35]. Advanced cognitive dispute procedures or behavioral experiments were introduced, e.g. "Anger chair" or "Guilt letter" assignments [24].

Fourth, attention was paid to existential issues such as the recurring thought that the patient could have died in the accident and if and how one could regard oneself as positively changed or personally grown by overcoming the traumatic experience and its aftermath [23]. When patients mentioned positive changes as a result of coping with the trauma, the reported benefits were appreciated and attributed as the patients' personal successes. In addition, the repeated completion of a questionnaire on posttraumatic growth [36] provided further occasions for discussing this issue.

## Results

### Initial treatment outcome

The primary analysis in this study is the comparison of CAPS scores for the two conditions from pre-treatment to post-treatment. The relevant mean scores are in Table 2 along with the means for the CBT condition available for the 3-month follow-up.

**Table 2:** Pre-treatment and post-treatment CAPS scores on all groups and 3-months follow-up CAPS scores on treated groups

**Group**	**Time**		
	**Pre-Tx**	**Post-Tx**	**3-months FU**^**1**^

CBT			
M	47.6	18.3	18.9
SD	19.1	18.8	23.8
Wait List			
M	41.8	35.2	-
SD	17.1	23.0	-

^1^reduced N = 17

A repeated measures ANOVA (Group × Time) revealed a significant main effect of Time (F [1, 40] = 63.79, p < 0.001, Cohen's d = 2.53) and a significant interaction of Group × Time (F [1, 40] = 25.81, p < 0.001, d = 1.61) but no main effect of Group. A between group comparison at post-test by ANCOVA (controlled for pre-values) revealed that the CBT condition showed greater PTSD symptom reduction than WLC (F [1, 39] = 23.47, p < 0.001, d = 1.55).

The intent-to-treat analysis including data from drop-outs revealed similar results, a main significant effect of Time (F [1, 42] = 51.0, p < 0.001, d = 2.20) and a significant interaction of Group × Time (F [1, 42] = 18.9, p < 0.001, d = 1.34). For controlling the gender imbalance of the two groups a repeated measures ANOVA (Group × Time) was performed only for women (19 in CBT and 13 in WLC) and revealed an even higher significant main effect of Time (F [1, 30] = 83.02, p < 0.001, Cohen's d = 3.33) and a significant interaction of Group × Time (F [1, 30] = 33.69, p < 0.001, d = 2.12) but no main effect of Group.

A second way to view these data is in terms of the categorical variable of whether participants changed diagnostic category or improved from PTSD/subsyndromal PTSD to non-PTSD. This could also be seen as a measure of clinically significant change. The categorical diagnostic data from before to after treatment are shown in Table 3.

**Table 3:** Clinical significance: Categorical diagnostic results for PTSD before and after treatment

**Condition**	**Pre-treatment diagnoses**	**Post-treatment diagnoses**	**Follow-up diagnoses**
CBT (N = 21)	PTSD (N = 12)	PTSD (N = 3)	PTSD (N = 4)
		Sub-PTSD (N = 2)	Sub-PTSD (N = 1)
		Non-PTSD (N = 7)	Non-PTSD (N = 7)
	Sub-PTSD (N = 9)	PTSD (N = 0)	PTSD (N = 0)
		Sub-PTSD (N = 2)	Sub-PTSD (N = 0)
		Non-PTSD (N = 7)	Non-PTSD (N = 9)
Wait List (N = 21)	PTSD (N = 10)	PTSD (N = 6)	-
		Sub-PTSD (N = 3)	-
		Non-PTSD (N = 1)	-
	Sub-PTSD (N = 11)	PTSD (N = 2)	-
		Sub-PTSD (N = 6)	-
		Non-PTSD (N = 3)	-

These data were analyzed with a 2 × 2 chi square test comparing status of full recovery (non-PTSD) in the CBT or control condition. It revealed that CBT was superior to Wait List (Chi^2 ^[1, N = 21] = 3.70, p < 0.05) post-treatment. Thus, in summary 67% of those with initial PTSD or sub-PTSD treated by CBT had improved, compared to 19% of those on the Wait List who were assessed twice.

Our assessments allowed us to examine possible changes in co-morbid major depressive disorder (MDD). It should be remembered that this co-morbid condition was not explicitly targeted by the treatment. Results for MDD were: Seven patients from CBT condition had MDD prior to treatment, all recovered from MDD at post-treatment. Three patients from WLC condition had MDD prior, 2 cases remained depressed by the post-treatment assessment. Chi squares test revealed, for those with MDD (49% of the sample), that CBT led to a significantly higher rate of recovery than Wait List (Chi^2 ^[1, N = 21] = 5.74, p < 0.05). Moreover, none of the participants in the CBT condition who were not depressed initially became depressed during treatment.

### Psychological questionnaires

Table 4 lists the pre-treatment and post-treatment values for each of the psychological tests (in the interest of brevity the 3-month follow-up values for the CBT participants are also listed). Also in the table there are (a) results for the Group × Time interaction from the Group × Time repeated measures MANOVA and (b) whether the within group change is significant from pre-treatment to post-treatment (and from post-treatment to 3-month follow-up).

**Table 4:** Psychological test results for all groups at pre-treatment, post-treatment and 3-months follow up

**Measure**		**Groups**		**Group × Pre-Post F value, effect size**	**ANCOVA post tests between groups with pretests as covariates/effect size**
	**Time**	**CBT**	**Wait List**		

IESR-Intrusion	Pre	21.7^a^	20.0^a^	F = 28.5, p < 0.001	F = 28.8, p < 0.001,
	Post	7.6^b^	17.6^a^	d = 1.89	d = 1.74
	FU	8.8^b^	-		
IESR-Avoidance	Pre	19.0^a^	16.5^a^	F = 10.7, p < 0.01	F = 10.4, p < 0.01
	Post	10.7^a^	16.6^a^	d = 1.16	d = 1.05
	FU	8.8^a^	-		
IESR-Hyperarousal	Pre	20.1^a^	20.7^a^	F = 11.9 p < 0.001	F = 8.9, p < 0.01
	Post	9.6^b^	16.6^a^	d = 1.22	d = 0.97
	FU	9.2^b^	-		
BDI	Pre	17.9^a^	16.0^a^	F = 4.9 p < 0.05	F = 6.6, p = 0.14
	Post	9.1^b^	12.6^a^	d = 0.78	d = 0.83
	FU	10.0^b^	-		
STAI	Pre	50.5^a^	49.1^a^	F = 6.4 p < 0.01	F = 6.5, p < 0.01
	Post	40.9^a^	46.2^a^	d = 0.89	d = 0.83
	FU	41.9^a^	-		
PTCI	Pre	123.3^a^	107.9^a^	F = 20.2 p < 0.001	F = 16.7, p < 0.001
	Post	86.1^b^	100.25^a^	d = 1.59	d = 1.33
	FU	92.5^b^	-		

^a, b, c ^Means within a column which share a superscript do not differ at p = 0.05

Examining the results in Table 4 one finds significant (all p < 0.01) change for those receiving CBT on measures of intrusive and avoidance symptoms, depressive symptoms (BDI), and posttraumatic cognitions (PTCI) from pre-treatment to post-treatment with no additional change in follow-up. Those in the Wait List condition did not significantly change on any measure. Between group comparisons at post-test by ANCOVAs (last column of table 4) revealed that the CBT condition showed greater change than WLC (all p < 0.01).

### Three-month follow-up outcome

For the 3-month follow-up, data of only n = 17 participants were available due to logistic problems of the research project. A series of repeated measure MANOVAs showed stability of measured psychopathology (see superscripts in table 4).

Table 2 already showed the mean CAPS scores at follow-up. Because of the lack of untreated control condition only tests of stability of treatment success in the CBT condition are available (paired t-test_post/FU(16) _= 0.7, p = 0.52). From post-treatment to follow-up, the recovery rate (i.e. rate of non-PTSD, see table 3) increased slightly from 67% to 76% of the initial CBT group.

## Discussion

This is the first German RCT utilizing a manualized CBT program for MVA survivors with PTSD. A combination of standard [8] and advanced cognitive-behavioral treatment (CBT) procedures led to significantly greater reductions on our chief dependent variable, the CAPS, in the CBT condition as compared to a Wait List control condition. Similar results were found for the categorical variable of whether participants continued to meet the symptomatic criteria for PTSD or not. When a more conservative re-analysis including drop-outs was performed, CBT remained superior to the Wait List.

These results are in accordance with previous findings which revealed that CBT is superior to control conditions (supportive psychotherapy, pharmacotherapy) in MVA survivors [14,37,38]. The degree of improvement in our CBT group was comparable to that in previously reported research trials [10,13] in finding treatment is superior to Wait List control condition. We acknowledge that other recently conducted treatment trials in other countries showed somewhat higher effect sizes for treatment outcomes [17-19]. These studies differ from the early CBT studies of PTSD treatment by introducing elaborated cognitive procedures to the treatment.

Our results extend those of the Blanchard and Hickling treatment study in two ways: the inclusion of advanced cognitive procedures to our treatment protocol (e.g., cognitive restructuring of guilt and anger, facilitating of posttraumatic growth) and our population had chronic PTSD since average interval between trauma and treatment was five years. Results were corroborated on several psychological questionnaires, including those related to PTSD, the IES-R and PTCI, the BDI for measuring depression, and the STAI for measuring anxiety.

There are several limitations of the current study. First, we were not able to systematically test if the newly added treatment techniques contributed to the general result over and above the CBT standard techniques. Further dismantling should clarify this question. Second, our study included a relatively large proportion of patients with subthreshold PTSD. Inclusion of subthreshold PTSD is partly justified by the high distress experienced by these cases [25] and the fact that ICD-10 criteria of PTSD as official reference for mental disorders definition in Germany has a lower diagnostic threshold than DSM-IV criteria. Third, although we applied a matching procedure in our randomization method there remained some initial group differences (e.g., gender imbalance, higher CAPS values of CBT group members) that may have resulted in underestimation of treatment success because not all participants could be matched into pairs according to gender, age and CAPS score. Furthermore, our random allocation procedure (matched pairs) appeared to have restricted random assignment slightly. And lastly, we only report short term (3 months) follow-up data here. This is to be resolved by reporting a longer-term follow-up in a subsequent paper.

## Conclusion

In conclusion, successful treatment procedures of PTSD are available in different languages. We had translated and somewhat supplemented a CBT protocol for the purpose of patients with German mother tongue. Thus, the current psychotherapy protocol seems suitable for PTSD patients. Furthermore, as recently discussed by other treatment researchers, both psychological and biological outcome measures can uncover more detailed information about the complexity of treatment response. Consequently, our research group will publish results of the presented treatment trial on changes in self-perceived posttraumatic growth [36] and on neurobiological changes initiated the present treatment protocol [e.g., [33]].

## Competing interests

The author(s) declare that they have no competing interests.

## Authors' contributions

AM planned the study, supervised the treatment, contributed substaintially to the statistical analyses and drafted the manuscript. TZ rephrased the treatment manual and carried out treatments. HM contributed to the data processing and performed statistical analyses. SR participated in treatments, data collection, and statistical analyses. AK conceived of the study, and participated in its design and coordination. All authors read and approved the final manuscript.

## Pre-publication history

The pre-publication history for this paper can be accessed here:


